# Inhibition of IL-6+IL-6 soluble receptor-stimulated aromatase activity by the IL-6 antagonist, Sant 7, in breast tissue-derived fibroblasts

**DOI:** 10.1038/sj.bjc.6600785

**Published:** 2003-02-18

**Authors:** A Purohit, A Singh, M W Ghilchik, O Serlupi-Crescenzi, M J Reed

**Affiliations:** 1 Endocrinology and Metabolic Medicine, Faculty of Medicine, Imperial College, St Mary's Hospital, London W2 1NY, UK; 2 The Breast Clinic, Central Middlesex Hospital, Acton Lane, Park Royal, London NW10 7NS, UK; 3 Department of Immunology (LABIO), Sigma-Tau S.p.A., Via Pontina Km 30,400, 00040 Pomezia (RM), Italy

**Keywords:** breast cancer, aromatase, cytokines, interleukin 6, prostaglandin E_2_, Sant 7

## Abstract

Interleukin 6 (IL-6) and its soluble receptor (IL-6sR) can markedly stimulate aromatase activity in cultured fibroblasts derived from normal or malignant breast tissues. IL-6 acts by binding to a low-affinity membrane-spanning receptor (IL-6R), which must associate with a high-affinity receptor (gp130) for signal transduction to occur. Sant 7 is a mutated form of IL-6 that can bind to the IL-6R, but inhibits its ability to interact with the gp130 signal transducing protein. In this study, we have used Sant 7 to examine its ability to inhibit IL-6+IL-6 soluble receptor (IL-6sR)-stimulated aromatase activity in breast tissue-derived fibroblasts. As previously observed, IL-6+IL-6sR markedly stimulated aromatase activity (7.7–20.8-fold) in fibroblasts derived from reduction mammoplasty tissue, tissue proximal to tumours and breast tumours. Sant 7 inhibited basal aromatase activity in some fibroblasts by 25–30% that had a high basal activity, but almost completely blocked the ability of IL-6+IL-6sR to stimulate aromatase activity. The IC_50_ for the inhibition of IL-6+IL-6sR-stimulated aromatase activity by Sant 7 was 60 ng ml^−1^. A comparison of the effects of prostaglandin E_2_ (PGE_2_), which can also regulate aromatase activity, and IL-6+IL-6sR revealed a greater degree of aromatase stimulation by IL-6+IL-6sR. Sant 7, however, inhibited PGE_2_-stimulated aromatase activity by 70% suggesting that PGE_2_ acts, in part, by stimulating IL-6 production. Much of the IL-6 and IL-6sR available to stimulate breast tumour aromatase activity may originate from infiltrating macrophages and lymphocytes. The ability to block aromatase stimulation by these factors may offer a novel therapeutic strategy for reducing oestrogen synthesis in breast tumours.

A number of potent aromatase inhibitors have now been introduced for use in postmenopausal women with hormone-dependent breast tumours (Miller, 1999). While their development represents an important advance in the therapies available for the treatment of women with breast cancer, the complete and partial response rates, when used as second-line therapy, remain relatively low (10–20%) with the time to tumour progression being relatively short (3–6 months) (Santen and Harvey, 1999). However, in a recent trial into the adjuvant use of an aromatase inhibitor *vs* tamoxifen, alone or in combination, disease-free survival was significantly longer for subjects receiving aromatase inhibitor therapy (ATAC Trialist Group, 2002). In addition, a number of adverse side effects, including gastrointestinal problems, dizziness and nausea, are associated with the use of some of the inhibitors (Buzdar *et al*, 1997). The use of aromatase inhibitors in postmenopausal women with breast cancer has also been reported to have an unfavourable effect on the serum lipid profile (Elisaf *et al*, 2001). There is, therefore, a need to develop new methods of inhibiting aromatase activity that may act specifically within the breast but spare other oestrogen-sensitive tissues.

The aromatase enzyme complex, which converts androstenedione to oestrone, has a pivotal role in controlling oestrogen synthesis in peripheral tissues in postmenopausal women. The enzyme is present not only in adipose tissue but also in normal and malignant breast tissues (James *et al*, 1987). Previous studies have revealed that the expression of the aromatase gene is regulated in a tissue-specific manner by the use of a number of different promoters (Mahendroo *et al*, 1993; Zhao *et al*, 1995a,1995b). In adipose tissue expression is regulated by promoter PI.4. The 5′-upstream region of this promoter contains a glucocortcoid response element and a GAS (IFN*γ* activating sequence) element, which can bind transcription factors of the signal transducer and activation of transcription (STAT) family (Zhao *et al*, 1995a,1995b). Cytokines in the presence of glucocorticoids regulate gene expression via PI.4. Promoter switching may occur in malignant breast tissues with an increase in the levels of PII and PI.3 being detected (Harada *et al*, 1993; Agarwal *et al*, 1996). Expression of the aromatase gene via PII and PI.3 is regulated by cyclic AMP (cAMP) and there is evidence that prostaglandin E_2_ (PGE_2_) may be the major factor regulating expression via these promoters (Zhao *et al*, 1996). Evidence has been obtained, however, to suggest that PGE_2_ may act, at least in part, to stimulate aromatase activity by induction of IL-6 (Zhang *et al*, 1988; Hinson *et al*, 1996).

Fibroblasts derived from normal or malignant breast tissues have been used as a model to investigate aromatase activity as these cells have a much higher level of activity than epithelial cells (Singh *et al*, 1999). Using such fibroblasts a number of cytokines including interleukin 6 (IL-6) and tumour necrosis factor *α* (TNF*α*) were identified as important regulators of fibroblast aromatase activity (Reed *et al*, 1992; Macdiarmid *et al*, 1994). Peripheral aromatase activity is increased in elderly and obese subjects (Grodin *et al*, 1973; Hemsell *et al*, 1974) and production of IL-6 is also increased in these conditions (Wei *et al*, 1992; Mohamed-Ali *et al*, 1997).

The ability of IL-6 to stimulate aromatase activity in cultured fibroblasts is markedly potentiated (up to 21-fold) by its soluble receptor, IL-6sR (Singh *et al*, 1995; Zhao *et al*, 1995a,1995b). IL-6 is produced by fibroblasts derived from normal and malignant breast tissues, whereas IL-6sR was only detected in conditioned medium collected from malignant fibroblasts (Singh *et al*, 1995). Many breast tumours are infiltrated by macrophages and lymphocytes and there is evidence that these cells may be a major source of factors that are able to stimulate aromatase activity within the breast (Purohit *et al*, 1995; Reed and Purohit, 1997).

Like other cytokines IL-6 acts by binding to a membrane-spanning receptor. The IL-6R complex consists of an 80 kDa (gp80) ligand-binding subunit and a 130 kDa (gp130) signal-transducing protein (Taga *et al*, 1989; Kishimoto *et al*, 1995). The gp80 subunit, which can also exist in a soluble form (Rose-John and Heinrich, 1994), binds IL-6 with low affinity and must associate with the larger gp130 for high affinity binding and signal transduction to occur. While IL-6 monoclonal antibodies (Mabs) have been used to abrogate the effects of IL-6, they are only partially effective (Klein *et al*, 1991). The Mabs form a complex with IL-6, which results in reduced clearance of the cytokine.

As an alternative approach to block the actions of IL-6 Ciliberto and colleagues generated a number of IL-6 receptor antagonists, which were mutated forms of IL-6, that can bind to the IL-6R but block it in an inactive configuration (Demartis *et al*, 1996). This inhibits its ability to interact with the gp130 signal-transducing protein. The IL-6 variant Sant 7 was identified as the most potent superantagonist and it was able to inhibit the IL-6-stimulated growth of several different malignant cell types. In view of the important role that IL-6+IL-6sR have in regulating aromatase activity, the ability of Sant 7 to block cytokine-stimulated aromatase activity has been investigated. Sant 7 was also employed to obtain further information about the process by which PGE_2_ regulates aromatase activity in breast tissue-derived fibroblasts.

## MATERIALS AND METHODS

### Culture of fibroblasts

Samples of breast adipose tissue were collected from women undergoing reduction mammoplasty. In addition, samples of breast adipose tissue proximal to breast tumours (i.e. ‘non-involved’ tissue) and breast tumours were collected from women undergoing lumpectomy or mastectomy. Tissue samples were collected after obtaining subjects' informed consent to the study, which was approved by the hospital Ethics Committee.

Resected tissues were minced with scalpels and incubated in Eagles' modified minimum essential medium (EMEM) for 18–24 h at 37° with collagenase (200 *μ*g ml^−1^). The dispersed cells were harvested by centrifugation and washed twice with medium to remove collagenase. Dispersed cells were seeded into 25 cm^2^ culture flasks and allowed to attach. Cells were grown to confluence in EMEM containing HEPES buffer (20 mmol l^−1^), 10% fetal calf serum (FCS) and supplements (Reed *et al*, 1992). Cells were routinely passaged two to three times after which replicate 25 cm^2^ culture flasks were seeded and grown to 70–80% confluency. The medium was replaced with 2%c. charcoal-stripped FCS, phenol red-free EMEM and treatments were added in this medium for 48 h in the presence of dexamethasone (100 nM) and included: IL-6+IL-6sR (50 and 100 ng ml^−1^, R&D Systems Ltd, Abingdon, Oxon, UK) and PGE_2_ (10 *μ*M, Sigma, Poole, Dorset, UK).

### Sant 7

The IL-6R superantagonist Sant 7 was obtained from Sigma-Tau (Rome, Italy) and synthesised as previously described (Salvati *et al*, 1995). Sant 7 was dissolved in culture medium before adding to cells.

### Aromatase assay

Aromatase activity was measured in intact fibroblast monolayers using (1*β*-^3^H) androstenedione (15–30 Ci mmol^−1^, NEN- Du Pont, Stevenage, Herts, UK) over a 3–20 h period (Reed *et al*, 1992). Briefly, fibroblast monolayers were washed once with Earle's balanced salt solution EM (2.5 ml) unless stated otherwise. To each flask ^3^H androstenedione (0.25 *μ*Ci) was added to give a final substrate concentration of 3–4 nM. Fibroblast monolayers were incubated with substrate for 3–20 h at 37°C depending on the basal aromatase activity in the cells. Flasks containing no cells were also incubated with substrate and serum-free medium as assay blanks. After incubation, an aliquot of medium (2 ml) was removed from each flask and aliquots were extracted twice with diethylether (5 ml), which was discarded. The remaining aqueous phase was treated with an equal volume of a solution containing charcoal (5.0%) and dextran (0.5%), centrifuged and an aliquot of the supernatant (1 ml) was taken to determine its radioactive content by liquid scintillation spectrometry. It has previously been established that aromatase activity, as measured in fibroblasts, is linear with respect to time for up to 24 h (Macdiarmid *et al*, 1994).

### Statistics

The significance of differences in aromatase activity in treated and control cells was assessed using Student's *t*-test. Representative example of results are shown for experiments that were repeated 2–3 times.

## RESULTS

**Figure 1:**
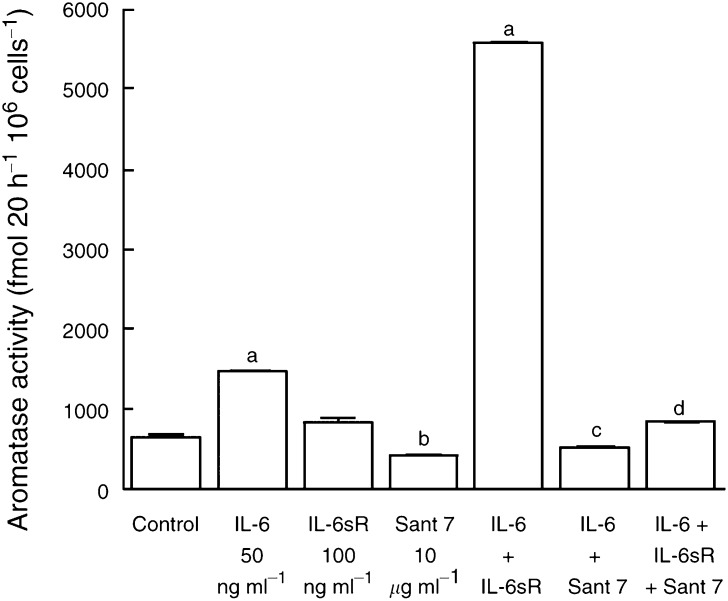
Effect of the IL-6 receptor superantagonist, Sant 7, on IL-6, IL-6sR or IL-6+IL-6sR-stimulated aromatase activity in fibroblasts derived from reduction mammoplasty breast adipose tissue. Fibroblasts were cultured in 2% stripped FCS, phenol red-free EMEM and treatments were added in this medium for 48 h in the presence of dexamethasone (100 nM). Aromatase activity was measured in intact fibroblast monolayers using [^3^H-1*β*]androstenedione as the substrate. The significance of differences in treated and control cells was assessed using Student's *t*-test (a, *P*<0.001 *vs* controls; b, *P*<0.05 *vs* controls; c, *P*<0.001 *vs* IL-6; d, *P*<0.001 *vs* IL-6+IL-6sR, means±s.d., *n*=3).

**Figure 2:**
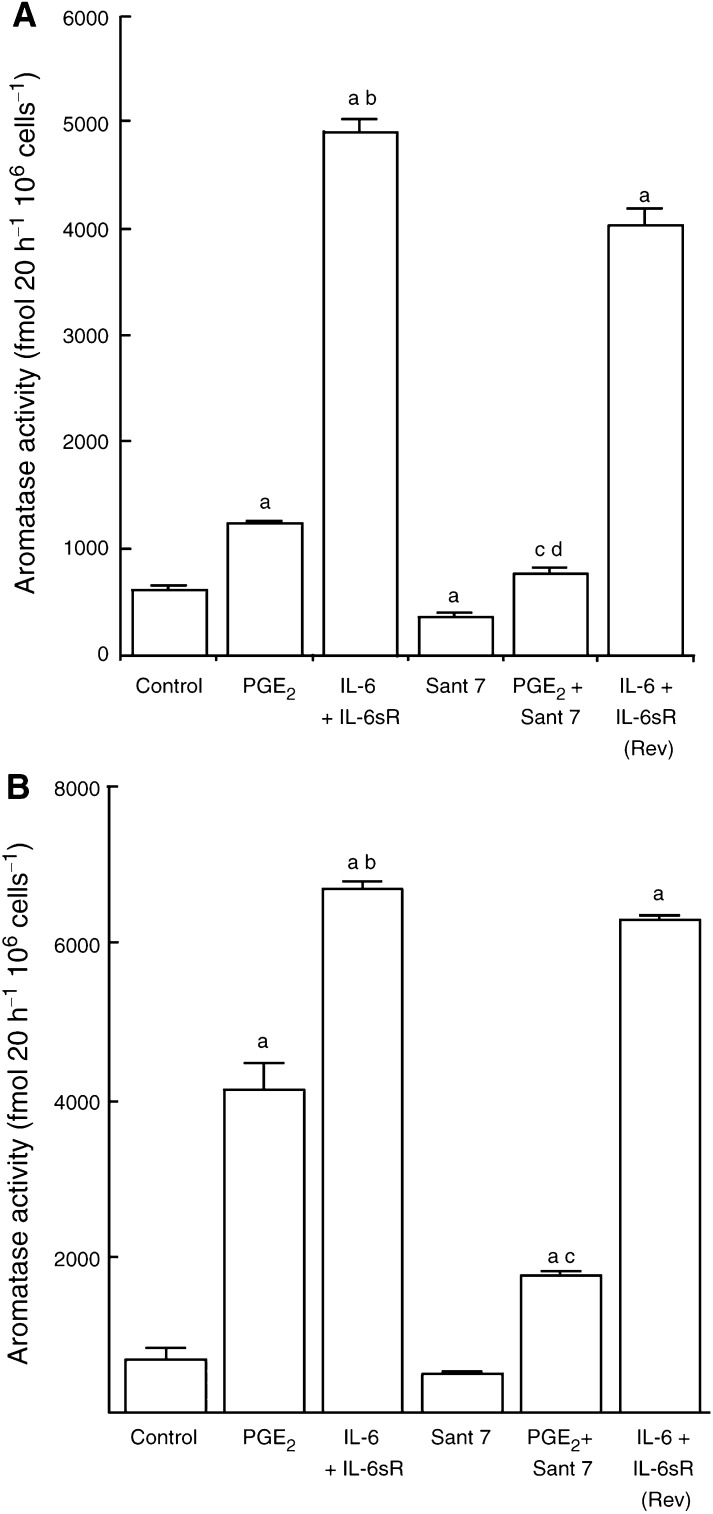
(**A**) Effect of Sant 7 on IL-6, IL-6sR or IL-6+IL-6sR-stimulated aromatase activity in fibroblasts derived from tissue proximal to a breast tumour. Fibroblasts were cultured in 2% stripped FCS, phenol red-free EMEM and treatments were added in this medium for 48 h in the presence of dexamethasone (100 nM). Aromatase activity was measured in intact monolayers using [^3^H-1*β*]androstenedione as the substrate. One set of cells (pretreatment) was preincubated with Sant 7 for 3 h before the addition of IL-6+IL-6sR, while for another set (no pretreatment) Sant 7 was added at the same time as IL-6+IL-6sR (a, *P*<0.001 *vs* controls; b, *P*<0.001 *vs* IL-6+IL-6sR, means±s.d., *n*=3). (**B**) Effect of Sant 7 on IL-6 or IL-6+IL-6sR-stimulated aromatase activity in fibroblasts derived from a breast tumour. Culture conditions were as described above (a, *P*<0.001 *vs* controls; b, *P*<0.001 *vs* IL-6+IL-6sR, means±s.d., *n*=3).

**Figure 3:**
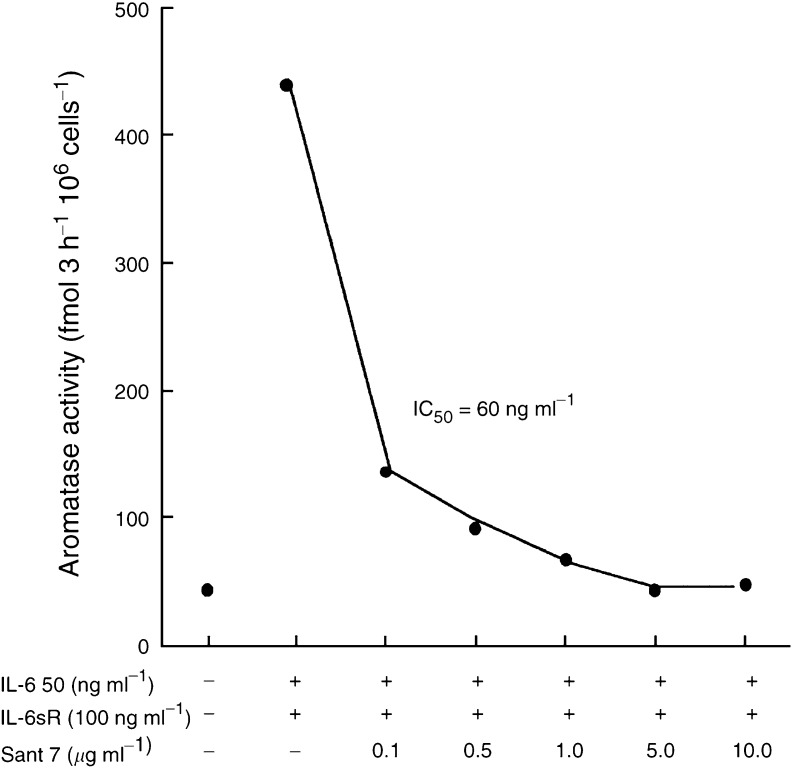
Dose response for the ability of Sant 7 to inhibit IL-6+IL-6sR-stimulated aromatase in fibroblasts derived from tissue proximal to a breast tumour. Sant 7 inhibited the stimulated aromatase activity with an IC_50_ of 60 ng ml^−1^ (means of triplicate measurements for which the coefficients of variation were <10%).

The ability of Sant 7 to inhibit cytokine-stimulated aromatase activity was initially examined in fibroblasts derived from breast adipose tissue of a subject undergoing reduction mammoplasty (Figure 1

). In these fibroblasts, IL-6 alone, (at 50 ng ml^−1^) increased aromatase activity by 27%. The addition of IL-6sR in combination with IL-6, however, markedly potentiated its ability to stimulate aromatase activity (7.7-fold compared with controls). Sant 7 caused a significant (*P*<0.05) decrease in basal aromatase activity and IL-6-stimulated activity (*P*<0.001). Sant 7 was able to almost completely block the ability of IL-6+IL-6sR to stimulate aromatase activity. In a further series of experiments, the ability of Sant 7 to block cytokine-stimulated aromatase activity in fibroblasts derived from tissue proximal to a tumour (proximal fibroblasts) and also the tumour (tumour fibroblasts) from the same subject was examined (Figures 2A and B

). In the presence of dexamethasone, basal aromatase activity was 10 times higher in proximal fibroblasts than in tumour fibroblasts. The extent to which IL-6+IL-6sR-stimulated aromatase was also greater in proximal fibroblasts (20.8-fold) than in tumour fibroblasts (7.9-fold). No apparent differences were detected in the cellular homogeneity or viability of fibroblasts derived from tumour or proximal breast tissues that might explain the marked differences in basal and IL-6+IL-6sR-stimulated aromatase activity in these different fibroblasts. In both types of fibroblasts Sant 7 completely blocked IL-6+IL-6sR stimulation of aromatase activity. Preincubation of proximal fibroblasts with Sant 7, prior to the addition of IL-6+IL-6sR, did not increase its ability to block aromatase stimulation (Figure 2A). A dose–response study was carried out using proximal fibroblasts. The IC_50_ was calculated as the concentration of Sant 7 that inhibited IL-6+IL-6sR-stimulated aromatase activity by 50% and was 60 ng ml^−1^ (Figure 3

).

**Figure 4:**
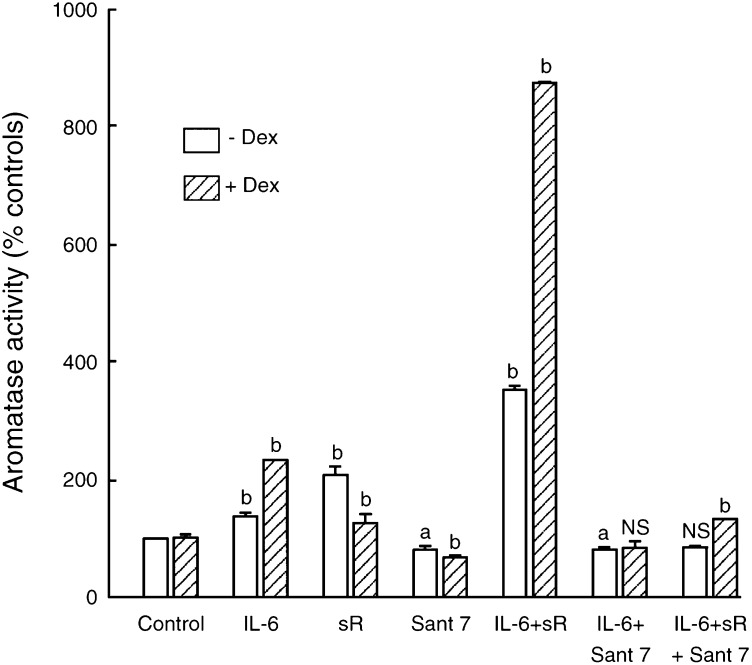
Ability of Sant 7 to inhibit IL-6+IL-6 sR stimulation of aromatase activity in fibroblasts derived from reduction mammoplasty tissue. Cells were treated for 48 h with IL-6 (50 ng ml^−1^), IL-6sR (100 ng ml^−1^) or Sant 7 (10 *μ*g ml^−1^), or in combinations as shown, in the absence or presence of dexamethasone (Dex, 100 nM). Aromatase activity in control cells was 1370±15 fmol 20 h^−1^ 10^6^ cells^−1^ and 639±35 fmol 20h^−1^ 10^6^ cells^−1^ for fibroblasts cultured in the absence or presence of dexamethasone (means±s.d., *n*=3; a, *P*<0.05 *vs* controls; b, *P*<0.001 *vs* controls; NS, not significant).

To examine if dexamethasone was an absolute requirement for the ability of Sant 7 to block IL-6+IL-6sR-induced aromatase activity, an experiment was carried out in the absence or presence of this glucocorticoid (Figure 4

). As shown, a similar pattern of responses to IL-6, IL-6sR or Sant 7, alone or in combination was seen in the absence or presence of dexamethasone. However, the extent of simulation by IL-6+IL-6sR was considerably greater (772%) in its presence than in its absence (252%). Sant 7 did inhibit the IL-6+IL-6sR-induced aromatase activity in the absence of dexamethasone.

**Figure 5:**
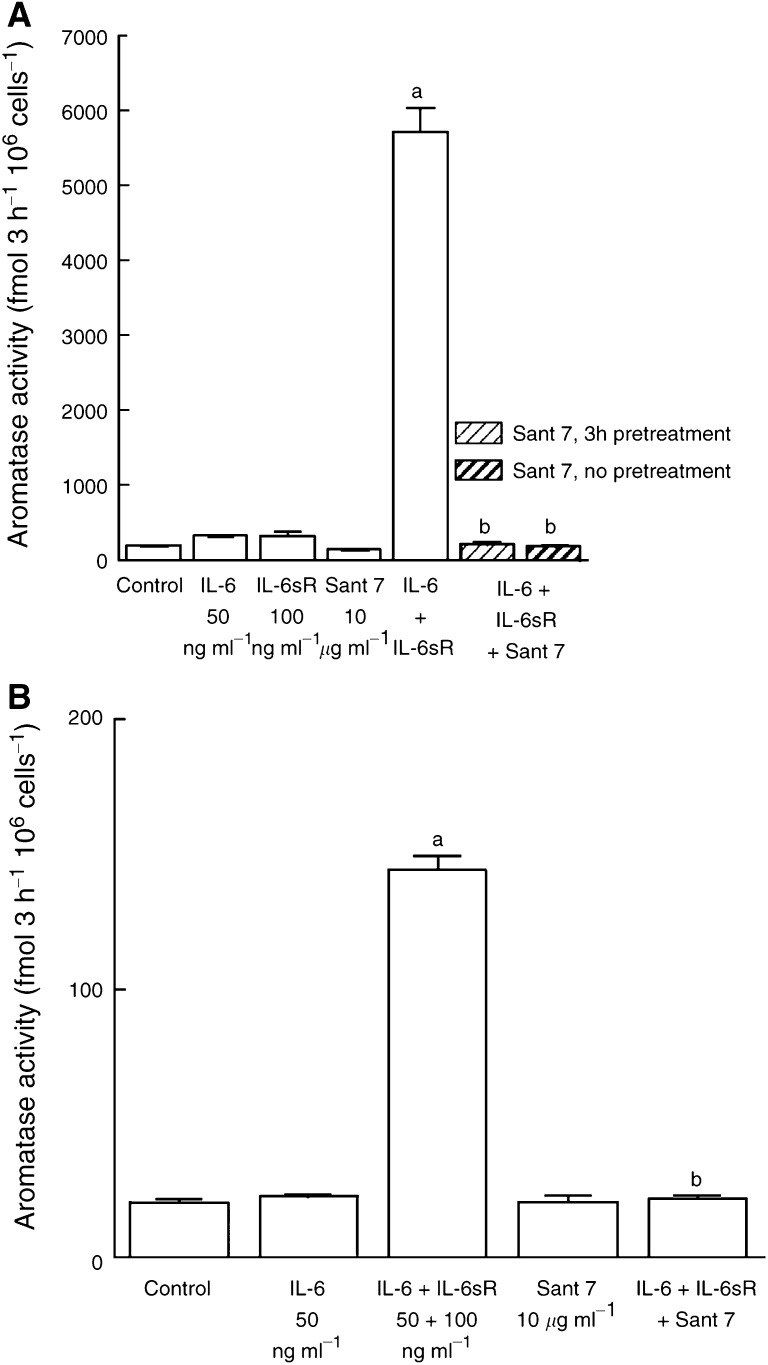
(**A**) Effect of Sant 7 on PGE_2_-stimulated aromatase in proximal fibroblasts. IL-6 (50 ng ml^−1^)+IL-6sR (100 ng ml^−1^) stimulated aromatase activity to a greater extent than did PGE_2_ (10 *μ*M). Sant 7, which blocks IL-6+IL-6sR-stimulated aromatase activity, significantly reduced the ability of PEG_2_ to stimulate aromatase activity in these fibroblasts. To examine if the effects of Sant 7 were reversible (Rev), one set of fibroblasts (IL-6+IL-6sR (Rev)) was preincubated with Sant 7 for 12 h and then Sant 7 was removed by washing the cells with phosphate-buffered saline. IL-6+IL-6sR stimulated aromatase activity in these cells after washing to a similar extent to fibroblasts not exposed to Sant 7, showing that Sant 7 does not act in an irreversible manner. (a, *P*<0.001 *vs* controls; b, *P*<0.0001 *vs* PGE_2_; c, *P*<0.001 PGE_2_+Sant 7 *vs* PGE_2_). (**B**) As for the legend to Figure 4A but carried out using breast tumour-derived fibroblasts (a, *P*<0.001 *vs* controls; b, *P*<0.001 *vs* PGE_2_; c, *P*<0.05 *vs* controls; d, *P*<0.001 *vs* PGE_2_, means±s.d., *n*=3).

The regulation of aromatase gene expression is complex and controlled by factors such as PGE_2_ and IL-6 (Mahendroo *et al*, 1993). There is evidence, however, that PGE_2_ may act, in part, to stimulate aromatase activity by the induction of IL-6 (Singh *et al*, 1999). As Sant 7 effectively blocks stimulation of aromatase activity by IL-6+IL-6sR, it was used to obtain further insight into its regulation by IL-6 or PGE_2_. In proximal and tumour fibroblasts, derived from the same subject, PGE_2_ stimulated aromatase activity by 521 and 103%, respectively (Figures 5A and B

). The combination of IL-6+IL-6sR was considerably more potent at simulating activity in proximal and tumour fibroblasts than PGE_2_, by 9.6– and 7.1-fold, respectively. In these fibroblasts Sant 7 itself inhibited basal aromatase activity by 30%. Sant 7 reduced the ability of PGE_2_ to stimulate aromatase activity in proximal and tumour fibroblasts by 69 and 75%, respectively. In these experiments the ability of Sant 7 to act in a reversible or irreversible manner was also examined (Figures 4A and B). Fibroblasts were preincubated for 12 h with Sant 7, after which it was removed from the cells by washing with phosphate-buffered saline. Subsequent addition of IL-6+IL-6sR showed that they were able to stimulate aromatase activity indicating that Sant 7 did not bind to the IL-6R in an irreversible manner.

## DISCUSSION

The results obtained from these studies have confirmed previous findings that IL-6+IL-6sR can markedly stimulate aromatase activity in breast tissue-derived fibroblasts (Singh *et al*, 1995; Zhao *et al*, 1995a,1995b). The potentiation by IL-6sR of the IL-6 stimulation of aromatase activity presumably results from an increase in the interaction of the IL-6–IL-6sR complex with the gp130 signal-transduction protein. In some cell systems the combination of IL-6+dexamethasone can markedly upregulate the expression of gp130 mRNA (Schooltink *et al*, 1992). In a previous study, the ability of Sant 7 to inhibit the proliferation of multiple myeloma cells was found to be dependent upon the presence of dexamethasone and retinonic acid (Honemann *et al*, 2001). This was not the case in the present study where Sant 7 was able to inhibit IL-6+IL-6sR-induced aromatase activity in the absence of glucocorticoid. The ability of IL-6+IL-6sR to stimulate aromatase activity was almost completely blocked by Sant 7 in all the fibroblasts examined. Sant 7 is a mutated form of IL-6 that binds to the IL-6R with an increased affinity that results in an inactive configuration of the receptor (Demartis *et al*, 1996). In addition to blocking cytokine-stimulated aromatase activity, Sant 7 also reduced the basal activity of this enzyme in some fibroblasts that had a relatively high basal activity, by up to 30%. It has previously been shown that breast tissue-derived fibroblasts can secrete IL-6 (Purohit *et al*, 1995). The finding that Sant 7 can reduce basal aromatase activity in these cells suggests that the IL-6 they produce is able to act in an autocrine/paracine manner to increase aromatase activity.

In related studies into the control of aromatase activity, the ability of a number of 10–16 amino-acid peptides to inhibit IL-6+IL-6sR-stimulated aromatase activity was previously examined (Parish *et al*, 2001). The 16 amino-acid peptide, AROHIB, at 10 *μ*M inhibited the ability of these cytokines to stimulate aromatase activity by 65%. AROHIB is therefore a less potent inhibitor of IL-6+IL-6sR-stimulated aromatase activity than Sant 7. Furthermore, to be effective it was necessary to preincubate cells with AROHIB prior to the addition of IL-6+IL-6sR. For Sant 7 no preincubation period was found to be necessary. Sant 7, however, does not bind to the IL-6R in an irreversible manner as preincubation of fibroblasts followed by washing with phosphate-buffered saline restored the ability of IL-6+IL-6sR to stimulate aromatase activity.

There is now good evidence that malignant fibroblasts produce IL-6 and IL-6sR and that tumour infiltrating macrophages and lymphocytes may also be an important source of factors that can stimulate oestrogen synthesis in breast tumours (Purohit *et al*, 1995; Singh *et al*, 1997). If IL-6 and IL-6sR, derived from these cells, are important regulators of aromatase activity, then the use of Sant 7 may offer a means of selectively blocking aromatase stimulation within the breast. Although small molecule-based aromatase inhibitors are being used for breast cancer therapy they can only be used in postmenopausal women. Their use in premenopausal women results in increased gonadotrophin production that overcomes the aromatase blockage. Thus, the ability to inhibit cytokine-stimulated aromatase activity in breast tissues of premenopausal women, either in the preventive or therapeutic setting, could be an important option for the use of Sant 7.

In addition to cytokines stimulating aromatase activity, PGE_2_ has also been implicated in the control of this enzyme (Zhao *et al*, 1996). However, determining the extent of regulation of aromatase activity by PGE_2_ in fibroblasts is complicated by the finding that PGE_2_, or factors that can increase intracellular cAMP levels, can stimulate IL-6 secretion by cells (Zhang *et al*, 1988; Hinson *et al*, 1996). Sant 7 was therefore employed in an attempt to determine whether PGE_2_ acts to stimulate aromatase activity by induction of IL-6. It was reasoned that if PGE_2_ is acting by the induction of IL-6, then Sant 7 should block, or reduce, its ability to stimulate aromatase activity. It has previously been shown that the ability of PGE_2_ to stimulate aromatase activity in breast tissue-derived fibroblasts is associated with a significant increase in IL-6 production by these cells (Singh *et al*, 1997). As consistently observed in previous studies, the ability of PGE_2_ to stimulate aromatase activity in proximal and tumour fibroblasts (520 and 100%, respectively) was considerably lower than that achieved with IL-6+IL-6sR (960 and 710%, respectively). Sant 7 reduced the PGE_2_ stimulation of aromatase activity by 69 and 75% in proximal and tumour fibroblasts, respectively. As Sant 7 only blocks IL-6-stimulated activity, this important finding indicates that a major part of the ability of PGE_2_ to stimulate aromatase activity results from its effect on IL-6 production.

IL-6 is a pleiotropic cytokine that has a number of important physiological functions (Van Snick, 1990). Excess production is associated with a number of pathological conditions including multiple myeloma (Ludwig *et al*, 1991). IL-6 is also known to have a role in regulating androgen receptor expression in an androgen-independent manner (Chen *et al*, 2000). As a result it may be involved in making prostate tumours resistant to endocrine therapy (Lin *et al*, 2001). By blocking the action of IL-6, Sant 7 has been shown to potentiate the sensitivity of the hormone-dependent prostate carcinoma cell line PC-3 to the cytotoxic effects of etoposide and cisplatin (Borsellino *et al*, 1999). The development of IL-6R superantagonists should allow the role of IL-6 in hormone-dependent and -independent conditions, such as breast and prostate cancer, to be elucidated and may lead to their use as novel therapeutic options for their treatment.

## References

[bib1] Agarwal V, Bulun SE, Leitch M, Rohrich R, Simpson ER (1996) Use of alternative promoters to express the aromatase cytochrome P450 (CYP19) gene in breast adipose tissues of cancer-free and breast cancer patients. J Clin Endocrinol Metab 81: 3843–3849 8923826 10.1210/jcem.81.11.8923826

[bib2] ATAC Trialist Group (2002) Anastrozole alone or in combination with tamoxifen *versus* tamoxifen alone for adjuvant treatment of postmenopausal women with breast cancer: first results of the ATAC randomised trial. Lancet 359: 2131–2139 12090977 10.1016/s0140-6736(02)09088-8

[bib3] Borsellino N, Bonavida B, Ciliberto G, Toniatti C, Travali S, D'Alessandro N (1999) Blocking signalling through the gp130 receptor chain by interleukin-6 and oncostatin M inhibits PC-3 cell growth and sensitizes the tumour cells to etoposide and cisplatin-mediated cytotoxicity. Cancer 85: 134–144 9921985

[bib4] Buzdar AU, Jones SE, Vogel CL, Wotter J, Plourde P, Webster A (1997) A phase III trial comparing anastrozole (1 and 10 milligrams), a potent and selective aromatase inhibitor, with megestrol acetate in postmenopausal women with advanced breast carcinoma. Cancer 79: 730–739 9024711

[bib5] Chen T, Wang LH, Farrar WL (2000) Interleukin 6 activates androgen receptor-mediated gene expression through a signal transducer and activator of transcription 3-dependent pathway in LNCap prostate cancer cells. Cancer Res 60: 2132–2135 10786674

[bib6] Demartis A, Bernassola F, Savino R, Melino G, Ciliberto G (1996) Interleukin 6 receptor superantagonists are potent inducers of human multiple myeloma cell death. Cancer Res 56: 4213–4218 8797594

[bib7] Elisaf MS, Bairaktari ETh, Nicolaides C, Kakaidi B, Tzallas CS, Katsaraki A, Pavlides NA (2001) Effect of letrozole on the lipid profile in postmenopausal women with breast cancer. Eur J Cancer 37: 1510–1513 11506958 10.1016/s0959-8049(01)00155-1

[bib8] Grodin JM, Siiteri PK, MacDonald PC (1973) Sources of estrogen production in postmenopausal women. J Clin Endocrinol Metab 36: 207–214 4688315 10.1210/jcem-36-2-207

[bib9] Harada N, Utsumi T, Takagi Y (1993) Tissue-specific expression of the human aromatase cytochrome P450 gene by alternative use of multiple exons 1 and promoters, and switching of tissue-specific exons 1 in carcinogenesis. Proc Natl Acad Sci USA 90: 11312–11316 8248245 10.1073/pnas.90.23.11312 PMC47972

[bib10] Hemsell DL, Grodin JM, Brenner PF, Siiteri PK, MacDonald PC (1974) Plasma precursors of estrogen II. Correlation of the extent of conversion of plasma androstenedione to estrone with age. J Clin Endocrinol Metab 38: 476–479 4815174 10.1210/jcem-38-3-476

[bib11] Hinson RM, Williams JA, Shacter E (1996) Elevated interleukin 6 is induced by prostaglandin E_2_ in a murine model of inflammation: possible role of cyclooxygenase 2. Proc Natl Acad Sci USA 93: 4885–4890 8643498 10.1073/pnas.93.10.4885 PMC39374

[bib12] Honemann D, Chatterjee M, Savino R, Bommert R, Gramatzki M, Dorken B, Bargou RC (2001) The IL-6 receptor antagonist Sant 7 overcomes bone marrow stromal cell-mediated drug resistance of multiple myeloma cells. Int J Cancer 93: 674–680 11477577 10.1002/ijc.1388

[bib13] James VHT, McNeill JM, Lai LC, Newton CJ, Ghilchik MW, Reed MJ (1987) Aromatase activity in normal breast and breast tumour tissues: *in vivo* and *in vitro* studies. Steroids 50: 269–279 3509763 10.1016/0039-128x(83)90077-6

[bib14] Kishimoto T, Akira S, Nagarzaki M, Taga R (1995) Interleukin-6 family of cytokines and gp130. Blood 86: 1243–1254 7632928

[bib15] Klein B, Wijdenes J, Zhang X-G, Jourdan M, Boiron J-M, Brochier J, Liautard J, Merlin M, Clement C, Mourel-Fournier B, Zhao-Liang L, Mannoni P, Sany J, Baitaille R (1991) Murine anti-interleukin-6 monoclonal antibody therapy for a patient with plasma cell leukaemia. Blood 78: 1198–1204 1715218

[bib16] Lin D-L, Whitney MC, Yao Z, Keller ET (2001) Interleukin-6 induces androgen responsiveness in prostate cancer cells through up-regulation of androgen receptor expression. Clin Cancer Res 7: 1773–1781 11410519

[bib17] Ludwig H, Nacnbaur DM, Fritz E, Krainer M, Huber H (1991) Interleukin-6 is a prognostic factor in multiple myeloma. Blood 77: 2794–2795 2043775

[bib18] Macdiarmid F, Wang D, Duncan LJ, Purohit A, Ghilchik MW, Reed MJ (1994) Stimulation of aromatase activity in breast fibroblasts by tumour necrosis factor *α*. Mol Cell Endocrinol 106: 17–21 7895904 10.1016/0303-7207(94)90181-3

[bib19] Mahendroo MS, Mendelson CR, Simpson ER (1993) Tissue-specific and hormonally controlled alternative promoters regulate aromatase P450 gene expression in human ovary and fetal tissue. J Biol Chem 268: 19463–19470 7690033

[bib20] Miller WR (1999) Biology of aromatase inhibitors: pharmacology/endocrinology within the breast. Endocrine Relat Cancer 6: 187–195 10.1677/erc.0.0060187 10731108

[bib21] Mohamed-Ali V, Goodrick S, Rawesh A, Katz DR, Miles JM, Yudkin JS, Klein S, Coppack SW (1997) Subcutaneous adipose tissue releases interleukin-6 but not tumour necrosis factor-*α*, *in vivo*. J Clin Endocrinol Metab 82: 4196–4200 9398739 10.1210/jcem.82.12.4450

[bib22] Parish D, Purohit A, Singh A, Rosankiewicz J, Ghilchik MW, Reed MJ (2001) Peptide inhibition of cytokine-stimulated aromatase activity in breast tissue fibroblasts. J Steroid Biochem Mol Biol 79: 165–172 11850221 10.1016/s0960-0760(01)00155-8

[bib23] Purohit A, Ghilchik MW, Duncan LJ, Reed MJ (1995) Aromatase activity and interleukin-6 production by normal and malignant breast tissues. J Clin Endocrinol Metab 80: 3052–3058 7559896 10.1210/jcem.80.10.7559896

[bib24] Reed MJ, Coldham NG, Patel SR, Ghilchik MW, James VHT (1992) Interleukin-1 and interleukin-6 in breast cyst fluid: their role in regulating aromatase activity in breast cancer cells. J Endocrinol 132: R5–R8 1564416 10.1677/joe.0.132r005

[bib25] Reed MJ, Purohit MJA (1997) Breast cancer and the role of cytokines in regulating estrogen synthesis: an emerging hypothesis. Endocrine Rev 18: 701–715 9331549 10.1210/edrv.18.5.0314

[bib26] Rose-John S, Heinrich PC (1994) Soluble receptors for cytokines and growth factors: generation and biological function. Biochem J 300: ;281–190 10.1042/bj3000281 PMC1138158 8002928

[bib27] Salvati AL, Lahm A, Paonessa G, Ciliberto G, Toniatti C (1995) Interleukin-6 (IL-6) antagonism by soluble IL-6 receptor *α* mutated in the predicted gp130-binding interface. J Biol Chem 270: 12242–12249 7744875 10.1074/jbc.270.20.12242

[bib28] Santen RJ, Harvey HA (1999) Use of aromatase inhibitors in breast cancer. Endocrine Relat Cancer 6: 75–92 10.1677/erc.0.0060075 10732791

[bib29] Schooltink H, Schmitz-Van der Leur H, Heinrich PC, Rose-John S (1992) Upregulation of the interleukin-6 signal transduction protein (gp130) by interleukin-6 and dexamethasone. Eur J Biochem 190: 79–83 10.1016/0014-5793(92)80552-r 1544406

[bib30] Singh A, Purohit A, Duncan LJ, Mokbel K, Ghilchik MW, Reed MJ (1997) Control of aromatase in breast tumours: the role of the immune system. J Steroid Biochem Mol Biol 61: 185–192 9365189

[bib31] Singh A, Purohit A, Ghilchik MW, Reed MJ (1999) The regulation of aromatase activity in breast fibroblasts: the role of interleukin-6 and prostaglandin E_2_. Endocrine-Relat Cancer 6: 139–147 10.1677/erc.0.0060139 10731102

[bib32] Singh A, Purohit A, Wang DY, Duncan LJ, Ghilchik MW, Reed MJ (1995) IL-6sR: release from MCF-7 breast cancer cells and role in regulating peripheral oestrogen synthesis. J Endocrinol 147: R9–R12 7490545 10.1677/joe.0.147r009

[bib33] Taga T, Hibi M, Hirata Y, Yamasaki K, Yasukawa K, Matsuda T, Hirano T, Kishimoto T (1989) Interleukin- 6 triggers the association of its receptor with a possible signal transducer protein. Cell 58: 573–581 2788034 10.1016/0092-8674(89)90438-8

[bib34] Van Snick J (1990) Interleukin- 6: an overview. Annu Rev Immunol 8: 253–278 2188664 10.1146/annurev.iy.08.040190.001345

[bib35] Wei J, Xu H, Davies JL, Hemmings GP (1992) Increase of plasma IL-6 concentrations with age in healthy subjects. Life Sci 51: 1953–1956 1453878 10.1016/0024-3205(92)90112-3

[bib36] Zhang Y, Lin J-X, Vilcek J (1988) Synthesis of interleukin 6 (interferon-beta 2/*β* cell stimulatory factor 2) in human fibroblasts is triggered by an increase in intracellular cAMP. J Biol Chem 263: 6177–6182 2452159

[bib37] Zhao Y, Agarwal VR, Mendelson CR, Simpson ER (1996) Estrogen biosynthesis proximal to a breast tumour is stimulated by PGE_2_ via cyclic AMP leading to activation of promoter II of the *CYP19* (aromatase) gene. J Clin Endocrinol Metab 137: 5739–5742 10.1210/endo.137.12.8940410 8940410

[bib38] Zhao Y, Mendelson CR, Simpson ER (1995a) Characterisation of the sequences of the human CYP19 (aromatase) gene that mediate regulation by glucocorticoids in adipose stromal cells and fetal hepatocytes. Mol Endocrinol 9: 340–349 7776980 10.1210/mend.9.3.7776980

[bib39] Zhao Y, Nichols JE, Bulun SE, Mendelson CR, Simpson ER (1995b) Aromatase P450 gene expression in human adipose tissue. J Biol Chem 270: 16449–16457 7608217 10.1074/jbc.270.27.16449

